# Factors that Predict Negative Results of QuantiFERON-TB Gold In-Tube Test in Patients with Culture-Confirmed Tuberculosis: A Multicenter Retrospective Cohort Study

**DOI:** 10.1371/journal.pone.0129792

**Published:** 2015-06-12

**Authors:** Yong-Soo Kwon, Yee Hyung Kim, Kyeongman Jeon, Byeong-Ho Jeong, Yon Ju Ryu, Jae Chol Choi, Ho Cheol Kim, Won-Jung Koh

**Affiliations:** 1 Department of Internal Medicine, Chonnam National University Hospital, Gwangju, Korea; 2 Department of Pulmonary and Critical Care Medicine, Kyung Hee University Hospital at Gangdong, School of Medicine, Kyung Hee University, Seoul, Korea; 3 Division of Pulmonary and Critical Care Medicine, Department of Medicine, Samsung Medical Center, Sungkyunkwan University School of Medicine, Seoul, Korea; 4 Division of Pulmonary and Critical Care Medicine, Department of Medicine, Ewha Medical Center and Ewha Medical Research Institute, Ewha Womans University School of Medicine, Mokdong Hospital, Seoul, Korea; 5 Department of Internal Medicine, Chung-Ang University School of Medicine, Seoul, Korea; 6 Department of Internal Medicine, College of Medicine, Gyeongsang Institute of Health Sciences, Gyeongsang National University, Jinju, Korea; University of Cape Town, SOUTH AFRICA

## Abstract

**Background:**

Interferon-γ release assays such as the QuantiFERON-TB Gold In-Tube Test (QFT-GIT) are designed to detect *Mycobacterium tuberculosis* infections, whether latent or manifesting as disease. However, a substantial number of persons with culture-confirmed tuberculosis (TB) have negative QFT-GITs. Information on host factors contributing to false-negative and indeterminate results are limited.

**Methods:**

A multicenter retrospective cohort study was performed with 1,264 culture-confirmed TB patients older than 18 years who were subjected to the QFT-GIT at one of the six hospitals between May 2007 and February 2014. Patients with human immunodeficiency virus infection were excluded. Clinical and laboratory data were collected in South Korea.

**Results:**

Of all patients, 87.6% (1,107/1,264) were diagnosed with pulmonary TB and 12.4% (157/1,264) with extrapulmonary TB. The rate of negative results was 14.4% (182/1,264). The following factors were highly correlated with false-negative results in the QFT-GIT: advanced age (age ≥ 65 years, odds ratio [OR] 1.57, 95% confidence interval [CI] 1.03–2.39), bilateral disease as determined by chest radiography (OR 1.75, 95% CI 1.13–2.72), malignancy (OR 2.42, 95% CI 1.30–4.49), and lymphocytopenia (total lymphocyte count < 1.0 × 10^9^/L, OR 1.86, 95% CI 1.21–2.87).

**Conclusions:**

Consequently, QFT-GIT results need to be interpreted with caution in patients with these host risk factors such as the elderly, bilateral disease on chest radiography, or malignancy, or lymphocytopenia.

## Introduction

Tuberculosis (TB) is a serious health threat worldwide, with an estimated 8.6 million incident cases and 1.3 million deaths in 2012 [1]. The early detection of this disease is critical to allow for early treatment and prevent further infection. However, a highly sensitive culture-dependent test used for the diagnosis of TB can take approximately 6 to 8 weeks to confirm due to slow mycobacterial growth [2, 3]. Another diagnostic method—sputum smear microscopy—is a rapid, simple, and inexpensive tool for the diagnosis of active TB. Although this method is highly specific in areas with a high TB prevalence, its sensitivity is low and varies in the range of 20–60% [4, 5]. Theoretically, TB nucleic acid amplification tests could detect even the presence of a single mycobacterial cell, but a meta-analysis revealed variable sensitivity (pooled sensitivity 85%, range 36–100%) of this test [6].

Immunologic tests, such as the tuberculin skin test (TST) and interferon-γ release assays (IGRAs), can help clinicians diagnose TB and latent *Mycobacterium tuberculosis* infection (LTBI) by demonstrating immunologic response to *M*. *tuberculosis* antigens. While these tests cannot differentiate active disease from latent infection, and cannot differentiate current and resolved infection, they can facilitate diagnostic decisions when used in conjunction with other clinical information [7, 8].

The QuantiFERON-TB Gold In-Tube Test (QFT-GIT, Cellestis/Qiagen, Carnegie, Australia) is one of several commercially available IGRAs [9, 10]. QFT-GIT uses an enzyme-linked immunosorbent assay (ELISA) to quantify the interferon-γ response of fresh whole blood to a cocktail of *M*. *tuberculosis* antigens. The cocktail represents three *M*. *tuberculosis* proteins, early secretory antigenic target-6 (ESAT-6), culture filtrate protein-10 (CFP-10), and TB7.7. These antigens are not present in any bacillus Calmette—Guérin (BCG) vaccine strain and are absent from most nontuberculous mycobacteria (NTM). As a result, QFT-GIT may be more specific in people who have received BCG or infected with certain NTM. In recent meta-analyses, pooled QFT-GIT sensitivity among persons with confirmed TB ranged from 65 to 84% with the lowest sensitivity among those with human immunodeficiency virus (HIV) infection [7, 8, 11]. As a result of inadequate sensitivity, IGRAs are considered insufficient to rule out active TB [12–22]. Identification of the host factors associated with false-negative and indeterminate QFT-GITs may improve their utility as diagnostic aids.

## Methods

### Study population

This is a retrospective review of patients without HIV infection who were older than 18 years, diagnosed with culture-confirmed TB at one of the six South Korean hospitals with >500 beds between May 2007 and February 2014, and who had QFT-GIT completed prior to receiving TB treatment. QFT-GIT is performed according to the discretion of the treating clinician. These patients were identified by positive results on TB culture from sputum, bronchial washes, body fluids, and tissue samples. The clinical, radiographic, and bacteriological status data were retrospectively collected from these patients. The presence of miliary TB and bilateral disease was determined by chest radiography. Heavy drinking is defined as the consumption of ≥ 40 g pure alcohol/day for men and ≥ 20 g pure alcohol/day for women[23]. HIV was detected by screening using Architect HIV Ag/Ab Combo Kit (Abbott Laboratories, Abbot Park, IL, USA) to determine the presence of HIV antibody and/or antigen and confirming using an additional HIV western blot test (HIV BLOT 2.2 Western Blot Assay, MP Diagnostics, Asia Pacific Pte Ltd., Singapore). With respect to co-morbid conditions, chronic pulmonary disease was defined as a non-infectious chronic pulmonary disease such as chronic obstructive pulmonary disease and asthma. Chronic kidney disease was defined as a disease causing a progressive loss of renal function. Chronic heart disease was defined as disease requiring the long-term use of cardiac medications, such as coronary artery disease, valvular heart disease, cardiomyopathy, and cardiac arrhythmias. Chronic liver disease was defined as a disease causing progressive destruction and regeneration of the liver parenchyma such as chronic viral hepatitis, alcoholic liver disease, and liver cirrhosis. Anemia was defined according to the World Health Organization guidelines as baseline hemoglobin content < 13 g/dL in men or < 12 g/dL in women [24]. Lymphocytopenia was defined as a blood lymphocyte count < 1.0 × 10^9^/L.

## QuantiFERON-TB Gold In-Tube Test

QuantiFERON-TB Gold In-Tube Test (Cellestis, Victoria, Australia) were performed according to the manufacturer’s instructions. Briefly, three blood collection tubes were used: Nil control tube (negative control without antigens or mitogen), control tube (positive control containing phytohemagglutinin), and TB antigen tube (containing a peptide cocktail simulating the *M*. *tuberculosis*-specific antigens ESAT-6, CFP-10, and TB7.7). The blood tubes were incubated for 20 h at 37°C. The IFN-γ concentrations were then measured by ELISA with an automated microplate processor (Evolis Twin Plus system; Bio-Rad Laboratories, Hercules, CA, USA).

The QFT-GIT results for each patient were interpreted according to the manufacturer’s criteria. Briefly, the QFT-GIT result was defined as positive if the IFN-γ level of Nil was ≤ 8.0 IU/mL and that of TB antigen minus Nil was ≥ 0.35 IU/mL and ≥ 25% of Nil value. Negative results was defined if the IFN-γ level of Nil was ≤ 8.0 IU/mL, that of Mitogen minus Nil was ≥ 0.5 IU/mL, and that of TB antigen minus Nil was < 0.35 IU/mL or < 25% of Nil value. The results were reported as indeterminate if the IFN-γ level of Nil was ≤ 8.0 IU/mL, that of TB antigen minus Nil was< 0.35 IU/mL or ≥ 0.35 IU/mL and < 25% of Nil value, and Mitogen minus Nil was < 0.5 IU/mL (positive control failure) or if the IFN-γ level of Nil was > 8.0 IU/mL (negative control failure).

### Statistical analyses

Values are expressed as medians and in the interquartile range (IQR), or as numbers (percentages) in the text and tables. Continuous comparison were performed using the Mann-Whitney *U* test, and categorical comparisons were performed using the Chi-squared or Fisher’s exact tests between variables in patients with positive and negative QFT-GIT results. The risk factors for negative and indeterminate QFT-GIT results were evaluated by univariate comparison of all clinical and laboratory variables in Table 1 between those with positive and those with negative QFT-GIT results, and between those with positive and those with indeterminate QFT-GIT results. Variables with p values < 0.2 were included in subsequent multiple logistic regression tests. In order to detect the existence of collinearity in the variables in multiple logistic regression tests, we measured the variance inflation factor (VIF) and factors with VIF > 5 were considered as a presence of collinearity. In regression analysis, variables to be maintained in the final model were selected by stepwise and backward regression; a p value < 0.05 were considered statistically significant. The data were analyzed using SPSS for Windows version 21.0 (SPSS, IBM, Armonk, NY, USA).

**Table 1:** Baseline characteristics of patients with tuberculosis.

Characteristic	N (%), Median (IQR)^a^	Total
Age, years	53.0 (35.0–69.0)	1,264
≥65	405 (32.0)	1,264
Sex, male	718 (56.8)	1,264
Body mass index, kg/m^2^	21.2 (19.0–23.1)	1,145
<18.5	220 (19.2)	1,145
Symptoms		
Cough	555 (44.0)	1,260
Sputum	415 (32.9)	1,260
Dyspnea	194 (15.4)	1,258
Fever	257 (20.4)	1,259
Hemoptysis	89 (7.1)	1,259
Weight loss	183 (14.5)	1,259
No symptoms	323 (25.7)	1,258
Ever smoked	479 (43.7)	1,096
Heavy drinking	124 (14.3)	865
Previous TB history	165 (13.1)	1,263
Smear-positive sputum	422 (37.8)	1,115
Cavity (or cavities) on chest radiograph	185 (14.7)	1,260
Bilateral disease	286 (22.7)	1,259
Miliary TB	48 (3.8)	1,260
Disseminated TB	57 (4.5)	1,259
Sites of disease		1,264
Pulmonary TB	1106 (87.5)	1,264
Extrapulmonary TB	158 (12.5)	1,264
ICU admission	30 (2.4)	1,245
Co-morbid conditions		
Chronic pulmonary disease	27 (2.1)	1,260
Chronic kidney disease	39 (3.1)	1,260
Chronic heart disease	85 (6.7)	1,260
Diabetes mellitus	203 (16.1)	1,260
Neurologic disease	42 (3.6)	1,260
Chronic liver disease	65 (5.2)	1,260
Rheumatologic disease	17 (1.3)	1,260
Malignancy	92 (7.3)	1,264
Immunosuppressive agent use	12 (1.0)	1,260
Laboratory findings		
Hemoglobin, g/dL	12.7 (11.2–13.9)	1,236
Anemia (hemoglobin content of <130 g/L in men or <120 /dL in women)	563 (45.6)	1,236
Lymphocyte, cells/mm^3^	1422.1 (939.5–1931.3)	1,178
Lymphocytopenia (<1.0 × 10^9^/L)	294 (25.0)	1,178
Platelet, × 10^9^/L	255.0 (198.0–318.0)	1,261
Glucose, mmol/L	107.0 (94.0–129.0)	1,121
Total protein, g/L	6.9 (6.2–7.4)	1,223
Albumin, g/L	3.8 (3.2–4.2)	1,232
CRP, mg/L	1.8 (0.3–6.1)	1,192

IQR, interquartile range; TB, tuberculosis; ICU, intensive care unit; CRP, C-reactive protein.

^a^The data are presented as medians (IQR) for age, body mass index, and hemoglobin and as n (%) for all other factors.

### Ethics statement

The Institutional Review Board of Chonnam National University Hospital approved this study and has given permission for it to be reviewed and published, including information obtained from patient records (IRB No. CNUH-2014-229). Informed consent was waived because of the retrospective nature of the study, and patient information was anonymized and de-identified prior to analysis.

## Results

Of the 9,181 patients suspected to have active TB, the following were excluded: those with negative results in TB culture in clinical specimen (n = 4,965), those not subjected to the QFT-GIT before the start of medication (n = 2,943), and those with confirmed HIV antibodies (n = 9). A total of 1,264 patients whose specimen cultures confirmed active TB infection, and who were tested with QFT-GIT prior to medication were included in this study.

Of the 1,264 culture-confirmed TB patients, 1082 (85.6%) had positive, 182 (14.4%) had negative, and 40 (3.2%) had indeterminate QFT-GIT results. The patients’ baseline characteristics are shown in Table 1.

In the univariate analysis for the predictive factors of negative QFT-GIT results, age ≥ 65 years and body mass index (BMI) < 18.5 kg/m^2^, presence of dyspnea, bilateral disease on chest radiography, and ICU admission when the test was performed were considered significant. Among the laboratory findings, anemia, lymphocytopenia, total protein, albumin,and C-reactive protein were significant. Among the co-morbid conditions, the presence of malignancy was significant. Multivariate analysis showed that age ≥ 65 years, bilateral disease, malignancy, and lymphocytopenia were significant predictive factors for negative QFT-GIT results (Table 2). There were no collinarities among variables in the multiple logistic regression tests (VIFs < 2.1).

**Table 2:** Predictors of negative QuantiFERON-TB Gold In-Tube Test results in patients with culture confirmed tuberculosis.

Patient characteristic	Positive^a^	Negative^a^	Univariate analyses		Multivariate logistic regression analyses	
	n = 1,082	n = 142	OR (95% CI)	p-value	OR (95% CI)	p-value
Age ≥ 65 years	318/1,082 (29.4)	68/142 (47.9)	2.21 (1.55–3.15)	<0.001	1.60 (1.05–2.43)	0.029
Body mass index < 18.5kg/m^2^	178/983 (18.1)	34/128 (26.6)	1.64 (1.07–2.50)	0.023		
Dyspnea	149/1,076 (13.8)	30/142 (21.1)	1.67 (1.08–2.58)	0.022		
Fever	205/1,077 (19.0)	37/142 (26.1)	1.50 (1.00–2.25)	0.050		
Bilateral disease	220/1,078 (88.4)	46/141 (32.6)	1.89 (1.29–2.77)	0.001	1.78 (1.14–2.76)	0.011
Miliary TB	35/1,079 (3.2)	8/141 (5.7)	1.79 (0.82–3.95)	0.149		
Disseminated TB	43/1,077 (4.0)	9/142 (6.3)	1.63 (0.78–3.41)	0.198		
ICU admission	19/1,066 (1.8)	8/141 (5.7)	3.32 (1.42–7.72)	0.005		
Diabetes mellitus	161/1,078 (14.9)	29/142 (20.4)	1.46 (0.94–2.27)	0.092		
Neurologic disease	36/1,078 (3.3)	9/142 (6.3)	1.96 (0.92–4.16)	0.080		
Malignancy	71/1,082 (6.6)	17/142 (12.0)	1.94 (1.11–3.39)	0.021	2.50 (1.33–4.60)	0.004
Anemia	453/1,058 (42.8)	84/139 (60.4)	2.04 (1.42–2.93)	<0.001		
Lymphocytopenia	225/1,004 (22.4)	48/134 (35.8)	1.93 (1.32–2.83)	0.001	1.91 (1.24–2.95)	0.003
Total protein, g/L	69 (63–74) (n = 1,046)	6.60 (5.90–7.25) (n = 137)	0.76 (0.63–0.93)	0.006		
Albumin, g/L	39 (33–43) (n = 1,054)	3.45 (2.80–4.00) (n = 138)	0.50 (0.39–0.65)	<0.001		
CRP, mg/L	18 (3–61) (n = 1,023)	3.34 (0.70–8.47) (n = 131)	1.05 (1.02–1.08)	0.002		

OR, odds ratio; CI, confidence interval; TB, tuberculosis; ICU, intensive care unit; CRP, C-reactive protein

^a^The data are presented as medians (IQR) for total protein, albumin, and CRP and as n (%) for all other factors.

The predictive factors for indeterminate QFT-GIT results were evaluated by grouping the QFT-GIT results into positive and indeterminate sets. Symptoms of dyspnea, bilateral disease, diabetes mellitus (DM), immunosuppressive agent use, and low albumin levels, were the independent predictive factors for indeterminate QFT-GIT results in patients with culture-confirmed TB (Table 3). There were no collinarities among variables in the multiple logistic regression tests (VIFs < 2.1).

**Table 3:** Predictors of indeterminate QuantiFERON-TB Gold In-Tube test results in patients with culture confirmed tuberculosis.

Characteristic	Positive^a^	Indeterminate^a^	Univariate analyses		Multivariate logistic regression analyses	
	n = 1,082	n = 40	OR (95% CI)	p-value	OR (95% CI)	p-value
Age ≥ 65 years	318/1,082 (29.4)	19/40 (47.5)	2.17 (1.15–4.10)	0.016		
Sex, female	472/1,082 (43.6)	22/40 (55.0)	1.58 (0.84–2.98)	0.158		
Dyspnea	149/1,076 (13.8)	15/40 (37.5)	3.73 (1.92–7.25)	<0.001	2.29 (1.08–4.85)	0.031
Weight loss	149/1,077 (13.8)	11/40 (27.5)	2.36 (1.16–4.83)	0.018		
Fever	205/1,077 (19.0)	15/40 (37.5)	2.55 (1.32–4.93)	0.005		
Bilateral disease	220/1,078 (20.4)	20/40 (50.0)	3.90 (2.06–7.38)	<0.001	3.38 (1.65–6.94)	0.001
Miliary TB	35/1,079 (3.2)	5/40 (12.5)	4.26 (1.57–11.53)	0.004		
Disseminated TB	43/1,077 (4.0)	5/40 (12.5)	3.44 (1.28–9.20)	0.014		
ICU admission	19/1,066 (1.8)	3/38 (7.9)	4.72 (1.34–16.71)	0.016		
Chronic renal disease	28/1,078 (2.6)	6/40 (15.0)	6.62 (2.57–17.04)	< 0.001		
Diabetes mellitus	161/1,078 (14.9)	13/40 (32.5)	2.74 (1.39–5.43)	0.004	2.58 (1.20–5.54)	0.015
Chronic liver disease	54/1,078 (5.0)	5/40 (12.5)	2.71 (1.02–7.19)	0.045		
Rheumatic disease	13/1,078 (1.2)	2/40 (5.0)	4.32 (0.94–19.78)	0.060		
Immunosuppressive agent use	9/1,078 (0.8)	2/40 (5.0)	6.25 (1.31–29.93)	0.022	9.34 (1.33–66.24)	0.025
Anemia	453/1,058 (42.8)	26/39 (66.7)	2.67 (1.36–5.26)	0.004		
Lymphocytopenia	225/1,004 (22.4)	21/40 (52.5)	3.83 (2.02–7.24)	<0.001		
Total protein, g/L	6.90 (6.30–7.40) (n = 1,046)	5.75 (5.23–7.10) (n = 40)	0.42 (0.30–0.58)	<0.001		
Albumin, g/L	3.90 (3.30–4.30) (n = 1,054)	2.85 (2.50–3.40) (n = 40)	0.20 (0.12–0.32)	<0.001	0.22 (0.13–0.39)	<0.001
CRP, mg/L	1.80 (0.30–6.10) (n = 1,023)	5.62 (1.91–14.01) (n = 38)	1.10 (1.06–1.15)	<0.001		

OR, odds ratio; CI, confidence interval; TB, tuberculosis; ICU, intensive care unit; CRP, C-reactive protein.

^a^The data are presented as medians (IQR) for total protein, albumin, and CRP and as n (%) for all other factors.

## Discussion

To our knowledge, this multicenter study that included 1,264 culture-confirmed TB patients is the largest to date evaluating host risk factors resulting in false negative QFT-GIT. We found that some host factors that can lead to low cell-mediated immune responses, such as old age, extensive disease, malignancy, and lymphocytopenia were independent risk factors.

Old age remains a known risk factor for false-negative IGRA results and the current study supports these findings [12–14, 17, 18, 21]. The IFN-γ response in the QFT-GIT was decreased in elderly patients (≥80 years) with culture-confirmed TB, and the median age of patients with negative results was also higher [17, 18]. Age was also an independent risk factor for negative results being observed in smear-positive pulmonary TB as well as for indeterminate or negative results in culture-confirmed TB [13, 14]. We observed that advanced age (≥65 years of age) is an independent predictive factor for negative QFT-GIT results in patients with active TB, which was consistent with the findings of previous studies. However, age was not a significant predictive factor for indeterminate results in multivariate analysis. Some studies about the predictors for indeterminate QFT-GIT results also did not show older age as an independent factor. Therefore further studies are needed to evaluate this issue.

The relationship between disease severity and IFN-γ production was also analyzed; decreased IFN-γ production in patients with severe TB was reported in both HIV-negative and HIV-positive patients [25]. More radiographically severe disease was also significantly associated with negative QFT-GIT results, which suggests that the low QFT-GIT sensitivity in patients with TB may be related to the immunological inability of the host to contain mycobacterial replication [20]. These results are consistent with those of our study, which showed bilateral disease as an independent predictive factor for negative QFT-GIT results, despite miliary TB being significant only in univariate analysis. Dyspnea was an independent predictor for the evaluation of indeterminate QFT-GIT results. This might also be explained by severity of disease since extensive disease could cause frequent dyspnea in patients with active TB.

Immunosuppression can be a risk factor for negative IGRA results in patients with TB infections, and several studies have confirmed these findings in patients with active TB [15, 16, 21, 26, 27]. However, inconsistencies were observed according to different definitions of immunosuppression, and smaller patient number may have limited these findings. Malignancy was a risk factor for decreased QFT-GIT sensitivity, a finding that is consistent with previous studies [15, 16, 26].

Our study found an association between DM and indeterminate QFT-GIT results in patients with culture-confirmed TB. The reported sensitivity of QFT-GIT in diabetic TB patients is variable. Although some studies reported high sensitivity of QFT-GIT in diabetic TB patients [15, 28, 29], a recent study demonstrated a decrease in sensitivity of QFT-GIT in diagnostic TB patients [30]. Some of the proposed causes of the high incidence of indeterminate results include delayed *M*. *tuberculosis*-specific IFN-γ production in the lymph nodes of diabetic mice, as measured by enzyme-linked immunospot (ELISPOT), and decreased levels of *M*. *tuberculosis*-specific antigen-stimulated IFN-γ production in the whole blood of DM patients with latent TB infection [31, 32].

Among laboratory characteristics, lymphocytopenia was an independent risk factor in this study. QFT-GIT depends on the elaboration of IFN-γ by T lymphocytes previously sensitized with *M*. *tuberculosis*-specific antigens after stimulation [10]. Therefore, lymphocytopenia can decrease the production of IFN-γ and can cause false negative QFT-GIT results [18, 20, 26]. ypoalbuminemia can reflect poor nutritional status in patients that may result in suppression of the systemic immune response. In our study, low serum albumin level was an independent predictive factor for indeterminate results in patients with active TB.

Our study has limitations. First, the data analysis was conducted retrospectively and quantitative QFT-GIT results were not collected. Second, the number of patients who received immunosuppressive drugs was rather small. Therefore the power of our study to detect the impact of these patients in QFT-GIT may be weak. Finally, further studies are necessary to determine whether all of the factors identified in our study also affect the results of ELISPOT-based assays.

In conclusion, here we identified some host factors such as advanced age, extensive pulmonary TB, malignancy, and lymphocytopenia that can potentially lead to negative QFT-GIT results in patients with culture-confirmed TB. Consequently, QFT-GIT results need to be interpreted carefully in patients with these host risk factors.
